# Advancing clinician–patient communication in academic hospitals: Insights from a multisite rapid qualitative analysis

**DOI:** 10.1002/jhm.70525

**Published:** 2026-10-25

**Authors:** Blair P. Golden, Monish A. Sheth, Sonia Dalal, James D. Harrison, Anita Hegde, Karnjit Johl, Patricia E. Litkowski, Satyam Nayak, Andrea Porrovecchio, Lucy Z. Shi, Hillary L. Western, Andrew A. White, Mark V. Williams, Angela Keniston

**Affiliations:** 1 Division of Hospital Medicine, Department of Medicine, University of Wisconsin-Madison School of Medicine and Public Health, Madison, Wisconsin, USA; 2 Division of Hospital Medicine, Department of Medicine, Baylor Scott and White Medical Center, Temple, Texas, USA; 3 Division of Hospital Medicine, Department of Medicine, Johns Hopkins University, Baltimore, Maryland, USA; 4 Division of Hospital Medicine, Department of Medicine, UCSF Medical School, San Francisco, California, USA; 5 Division of Hospital Medicine, Department of Medicine, UT Southwestern, Dallas, Texas, USA; 6 Division of Hospital Medicine, Department of Medicine, University of California-Davis School of Medicine, Sacramento, California, USA; 7 Division of Hospital Medicine, Department of Medicine, Washington University School of Medicine, Saint Louis, Missouri, USA; 8 White Plains Hospital, Montefiore Hospital System, White Plains, New York, USA; 9 Division of Hospital Medicine, Department of Medicine, University of Colorado Anschutz School of Medicine, Denver, Colorado, USA; 10 Division of Hospital Medicine, Department of Medicine, University of Washington School of Medicine, Seattle, Washington, USA; 11 Division of General Medicine and Population Science, Department of Medicine, University of Alabama-Birmingham, Birmingham, Alabama, USA

## Abstract

**Background:**

High-quality clinician-patient communication is associated with improved patient understanding, trust, and healthcare outcomes. However, the hospital environment poses unique challenges to effective communication, and research documents that many hospitalized patients report poor communication with clinicians.

**Objective:**

Characterize current efforts and barriers to improving clinician-patient communication across hospital medicine.

**Methods:**

We conducted a multi-methods study using virtual focus groups with an online survey. Focus groups were held with hospital medicine clinicians and members of the Hospital Medicine ReEngineering Network (HOMERuN) Patient and Family Advisory Council (PFAC).

We analyzed focus groups using rapid qualitative analysis.

**Results:**

Across 8 total focus groups, 37 clinicians and 4 PFAC members participated. We received 26 survey responses (63% response rate); the majority (19, 73%) reported their institution had initiatives in place to improve clinician-patient communication. Our qualitative analysis revealed four primary themes: (1) hospital medicine communication initiatives vary widely in approaches and objectives; (2) trainees are taught foundational communication skills through formal and informal mechanisms, but these efforts may be siloed from other initiatives; (3) hospital medicine clinicians perceive multiple barriers to improving individual communication behaviors; and (4) systems-level factors make it challenging to effectively develop, evaluate, and sustain communication initiatives.

**Conclusions:**

Hospital medicine groups are actively engaged in diverse communication initiatives, but these efforts are undermined by fragmentation, inadequate feedback mechanisms, and competing priorities. Our findings highlight a need for coordinated strategies that align communication projects with real-world clinical workflows, provide clinicians with actionable feedback, and incorporate genuine partnership with patients and caregivers.

## INTRODUCTION

Extensive research documents that many hospitalized patients report poor communication with clinicians. A survey of over 10,000 hospitalized patients found that nearly half experienced a problem during their hospitalization; 31% did not always feel comfortable speaking up.^1^ Another study found that most hospitalized adults were unaware of their main doctor, and approximately a quarter received mixed messages from their care team.^2^ Communication issues also occur during the precarious hospital discharge care transition: in a study involving direct observations on the day of hospital discharge, most patients received no verbal instruction on post-hospital disease self-management or red-flag symptoms warranting follow-up.^3^

Patient comprehension, trust, and clinical outcomes are enhanced with effective clinician-patient communication.^4–6^ Hospitalized patients and caregivers identify transparent communication as essential for earning their trust.^7^ Furthermore, communication breakdowns may contribute to adverse outcomes, including medical errors and financial penalties due to poor performance on the Hospital Consumer Assessment and Healthcare Providers and Systems (HCAHPS) survey.^8^ Communication errors have dire patient safety consequences and are the most commonly attributable root cause for adverse events.^9^ Unfortunately, the hospital setting presents unique challenges for clinician-patient communication, including patients’ acute and rapidly changing clinical conditions, large and complex care teams, and frequent clinician hand-offs.^2^

Hospital clinicians and administrators have implemented numerous interventions to improve clinician-patient communication, including whiteboards, bedside rounding models, and structured communication frameworks.^10–12^ However, implementation of communication interventions, like many interventions in the hospital setting, is inconsistent across services and hospitals. Additionally, the perspectives of hospital medicine clinicians may not be considered in these efforts.^13^ To guide future research and quality improvement efforts, we aimed to characterize hospital medicine clinician-patient communication initiatives and barriers to implementation across a diverse range of academic hospital medicine programs.

## METHODS

### Study design, setting, and participants

We conducted a multi-methods study using focus groups with an online survey to characterize current hospital medicine efforts and barriers to improving clinician-patient communication. Focus groups were held on March 14, 2025, as part of the Hospital Medicine ReEngineering Network (HOMERuN) virtual monthly meetings. HOMERuN is a national collaborative of hospital medicine clinicians and researchers that aims to measure and improve the quality of care for hospitalized patients.^14^ This project was determined to be exempt by the UCSF Human Research Protection Program (IRB # 22–36424).

HOMERuN announces monthly topics in advance through email listservs, which include hospital medicine clinicians and members of the HOMERuN Patient and Family Advisory Council (PFAC), allowing members to identify sessions of interest. Clinician participants were randomly assigned to one of eight focus groups at the beginning of the meeting. We intentionally distributed PFAC members across separate focus groups to ensure that non-clinical perspectives were represented. Verbal consent was obtained prior to focus group recordings. No participation incentives were provided.

### Focus groups and an online survey

Each focus group had approximately five participants and lasted 30 min. Focus groups were moderated by study authors (B.P.G., M.S., S.D., L.S., J.H., K.J., A.W., A.K.) who are hospital medicine clinicians or researchers with training on conducting focus groups. Focus groups were recorded via Zoom, and moderators took field notes during focus groups. We asked participants to complete a 5-question REDCap survey concurrently with focus groups containing questions regarding participant demographics and current communication initiatives at their institution (Supporting Information S1: Supplement 1).^15,16^ We conducted a group debrief with all participants immediately following the focus groups.

Our interview guide (Supporting Information S1: Supplement 2) and survey were developed by study authors and refined with input from other HOMERuN working groups. Our interview guide explored hospital-based initiatives to improve clinician communication with patients, including efforts directed at trainees and hospital medicine clinicians. Respondents were asked to focus on initiatives that emphasized direct patient communication, rather than interdisciplinary, non–patient-facing communication. Participants were also asked to describe the extent to which patient and caregiver voices were incorporated into these initiatives and perceived implementation barriers.

### Analysis

We analyzed data using a rapid qualitative analysis approach, which uses templated summaries and matrix analyses instead of line-by-line coding to identify themes.^17,18^ We selected rapid qualitative analysis because it generates timely, actionable insights regarding implementation in complex systems, including hospitals.^18^ Team members first reviewed a recording of the same focus group and created a templated summary, which was reviewed as a group to resolve any discrepancies, including the level of detail. Individual team members (M.S., S.D., J.D.H., K.J., P.L., S.N., A.P., L.S., H.W., A.W., A.K.) then created a templated summary for each of the remaining focus groups. Each summary was reviewed by a team member (B.P.G.) to ensure consistency. We organized templated summaries into a matrix, with columns reflecting different domains within our interview guide and each row representing a unique focus group.^18^ We independently reviewed matrices to identify recurring patterns and any areas of discrepancy that occurred across rows, down columns, and diagonally, and created brief summaries of findings.^18^ We then met as a group to resolve discrepancies and discuss themes and sub-themes, which were refined through iterative discussions. We reviewed original recordings, field notes, and matrix summaries to verify the accuracy of our findings and further refine themes and sub-themes. We analyzed PFAC member contributions separately to ensure that non-clinical voices were included in our analysis. We conducted member checking with two participant authors (M.W., A.H.) and PFAC members who reviewed our findings to ensure that they reflected their experience as focus group members.

Survey responses were analyzed using descriptive statistics. Survey data were not linked to participants at the individual level; rather, they were used to provide contextual information about participants and institutional communication initiatives.

## RESULTS

Across 8 focus groups, there were 41 participants, including 4 PFAC members. We identified four primary themes: diverse communication initiatives, siloed communication training, barriers to improving individual communication behaviors, and system-level challenges to communication initiatives. Themes, sub-themes, and representative quotes are shown in Table 1.

We received 26 survey responses (63% response rate), most of which were from attending physicians (24, 92%). As shown in Table 2, respondents were represented from all seven geographic regions in the United States. The majority (19, 73%) reported that their institution has initiatives in place to improve clinician-patient communication. Respondents reported communication initiatives across a range of focus areas, consistent with the diversity of initiatives described during focus groups.

### Theme 1: Hospital medicine communication initiatives vary widely in approaches and objectives

#### Sub-theme 1a: Numerous and diverse initiatives

Participants described many different institutional initiatives to improve hospital medicine clinician-patient communication. While the specifics of initiatives varied, there were common areas of focus. For example, multiple participants described initiatives intended to increase patient recognition of their hospital team or understanding of their treatment plans. There were numerous tools used for implementation, including whiteboards, patient portals (e.g., MyChart), and printouts highlighting daily care plans (e.g., planned tests, medication changes, consultants). Multiple groups used “face cards” to facilitate recognition of clinicians and understanding of team roles. Others described initiatives focused on interdisciplinary bedside rounding to improve perceptions of collaborative care. Multiple participants described patient-facing initiatives focused on discharge coordination and education. Several described initiatives to promote discussion and clarification of patient care preferences and goals. Many of these programs were based on existing frameworks or best practices (e.g., Vital Talk). Some participants described initiatives and training to improve communication skills outside of goals of care conversations; examples of such programs included AIDET^®^ (Acknowledge, Introduce, Duration, Explanation, and Thank) training, “Commit to Sit” initiatives, and a curriculum to build skills with trauma-informed care.^19^ Finally, participants described initiatives focused solely on reviewing HCAHPS scores.

Across all initiatives, the level of feedback provided to hospital medicine clinicians was variable; some described programs providing hospital medicine clinicians individual feedback, whereas some initiatives provided no feedback. Others described receiving feedback via HCAHPS, including individual or group scores or narrative comments.

#### Sub-theme 1b: Varied objectives of communication initiatives

Participants also described varied motivations and objectives for communication initiatives. For example, some participants described initiatives that were primarily intended to improve patient comprehension, address health literacy barriers, or empower patients to ask questions about their care. Many participants related that the perceived objective of communication initiatives was to increase patient experience scores (i.e., HCAHPS).

#### Sub-theme 1c: Variable degree of engagement across institutions

Participants also described variable levels of institutional engagement in communication initiatives. For example, some relayed that their institution was actively engaged in multiple projects, whereas others were not aware of any current or recent initiatives. Some participants described a lack of sustained institutional engagement over time despite initial interest in the development of communication initiatives.

### Theme 2: Foundational communication skills taught through formal and informal mechanisms, but may be siloed from other initiatives

#### Sub-theme 2a: Formal and informal initiatives with variable feedback

Participants described both formal and informal communication training initiatives for undergraduate medical education and graduate medical education (GME) learners. Formal curricula on communication skills were commonly simulation-based, but had a range of foci, including more high-stakes conversations (e.g., breaking bad news) and more mundane communication skills. Some participants perceived that communication training for GME (resident) learners was less formalized and more “built into their expectations.” Some participants conveyed a need for a more robust curriculum focused on communication skills (particularly for GME learners), although one participant expressed concern that formal communication frameworks may not adequately prepare trainees for real-world practice. Others described informal initiatives to ensure that trainees receive feedback on communication skills during daily rounding. In contrast, one participant described a resource-intensive, formalized program at their internal medicine residency that included having a clinical psychologist observe residents and provide formal, structured feedback on their communication skills.

#### Sub-theme 2b: Siloing between trainee and hospital medicine clinician initiatives

A number of participants reflected that communication initiatives for trainees were generally separate from those intended for hospital medicine clinicians. Some participants gave examples of trainees not being included in concurrent initiatives for hospital medicine clinicians. Multiple participants commented that they were simply unaware of what might be happening in the educational sphere. Participants did not generally describe the siloing of trainee and hospital medicine clinician initiatives as problematic, noting that it primarily reflected different leadership within education and clinical domains.

### Theme 3: Hospital medicine clinicians perceive multiple barriers to improving individual communication behaviors

#### Subtheme 3a: Lack of meaningful, accessible metrics and feedback

Participants commonly expressed frustration with the perceived lack of meaningful, accessible metrics to provide real-time, actionable feedback at the individual level and evaluation of communication initiatives overall. Numerous participants reflected that HCAHPS metrics have uncertain relevance to clinicians, are not easily attributed to individuals, and may not accurately reflect communication quality. Participants also recognized that communication is a broad topic, meaning that a single evaluation tool may not be appropriate. Some reflected that monitoring rates of communication tasks (such as code status discussions) may paradoxically decrease the quality of communication by causing individuals to focus on “checking the box” rather than having a meaningful conversation. Participants also identified other barriers to providing feedback, including clinician sensitivity to criticism and the time and resources required to observe and coach individuals. In one focus group, participants discussed using technology to overcome some of these barriers; for example, ambient listening may facilitate clinician-level feedback on communication skills.

#### Subtheme 3b: Communication behaviors are challenging to change

Participants conveyed that communication skills are particularly difficult to modify despite targeted interventions. Multiple focus groups discussed potential generational differences in communication behaviors; some felt that senior and mid-career physicians may be reticent to change behaviors, whereas others mentioned that younger clinicians may be less inclined to spend time with patients at the bedside. Others conveyed a perception that communication skills may be more of an innate (rather than developed) skill or that lived experience (rather than training) shape communication behaviors.

#### Subtheme 3c: Competing demands and priorities

Participants described numerous competing demands and priorities in daily workflows that make it challenging for individuals to change their communication behaviors. Clinicians described workload factors (e.g., high patient volumes, pressure to discharge patients, frequent electronic chats) that made it difficult to spend time communicating directly with patients. Several focus groups discussed the potential utility of using patient portals to efficiently communicate with patients and overcome some of these barriers.

### Theme 4: Systems-level factors make it challenging to effectively develop, evaluate, and sustain communication initiatives

#### Subtheme 4a: Limited opportunities for patient and caregiver involvement in initiative development

Although we asked about efforts to include patients’ and family caregivers’ perspectives in communication initiatives, participants did not identify examples of initiatives that were co-developed with them. Participants described initiatives that were developed “for” patients rather than with patients. While most survey respondents reported having a PFAC at their institution, most participants did not identify direct links between their PFAC and communication initiatives. Furthermore, participants identified multiple barriers to engaging PFACs for communication projects, including hospital medicine divisions having limited access to PFACs and the administrative burdens of working with PFACs. One participant mentioned that their division was working to develop a hospital-medicine specific PFAC to overcome some of these barriers. One of the PFAC members noted that their institution recently downsized the patient advisory council due to financial considerations, therefore impacting the ability for patients to co-develop communication initiatives.

#### Subtheme 4b: Root causes of poor communication

Participants reflected that there were many root causes of poor communication between clinicians and patients. For example, multiple participants observed that patients may have mistrust of the healthcare system and that initiatives may not address these concerns. Others commented that there were broader system-level factors contributing to poor communication. For example, hospitalized patients often interact with multiple clinical teams, experience frequent hand-offs, and need specialty follow-up after hospitalization, all of which made communication challenging.

#### Subtheme 4c: Dispersed, fragmented initiatives with variable buy-in and leadership

Participants noted that communication initiatives often required customization to individual clinicians, hospital systems, or patient populations, leading to initiatives that were fragmented and not easily scalable. Participants observed that communication initiatives compete with multiple other quality initiatives, undermining clinician buy-in and sustainability. Communication initiatives may require resources that detract from other potential initiatives or profitability, making it challenging to obtain institutional buy-in. Participants also perceived barriers to obtaining buy-in for trainee initiatives, noting that communication skills are not heavily emphasized on standardized testing or learner evaluations, including the ACGME milestones. Multiple initiatives were described as driven by departmental or hospital-level leadership rather than hospital medicine clinicians themselves; participants reflected that leadership and clinicians may not be aligned on the motivations for such initiatives, which could compromise clinician buy-in and lead to initiatives that do not address meaningful communication barriers.

### PFAC member contributions

PFAC members primarily reflected on lived experiences of communication rather than commenting on specific institutional initiatives. They recalled difficulties communicating with clinicians during their own and family members’ illnesses. Participants described specific communication challenges arising during hospitalization, including experiencing confusion due to acute illness, large and transient clinical teams, and clinicians asking repetitive questions. PFAC members also identified several opportunities to improve hospital communication, including ensuring that team members communicate and share information, keeping communication simple and honest, using techniques such as teach-back, and including caregivers on rounds.^20^ PFAC members also emphasized the importance of approaching communication as a “conversation,” with input from both patients and clinicians: “You are not just caring for us, you are caring with us” (Group 5).

## DISCUSSION

In this multi-institutional qualitative study, hospital medicine clinicians described numerous, heterogenous communication initiatives that were implemented with variable objectives, engagement, and feedback mechanisms. While there is consistent evidence that hospitalized patients frequently experience communication problems across multiple domains (including diagnosis explanation, care team identification, and care transitions), our findings suggest that efforts to address these issues are not uniform or widespread. While learners receive training on foundational communication skills, this training may be disconnected from the systems in which clinicians ultimately practice. Finally, hospital medicine clinicians identified numerous barriers that undermine the effective development, evaluation, and sustainability of communication interventions, including the difficulty addressing root causes of poor communication, insufficient and imperfect feedback metrics, competing demands, and limited resources.

A common frustration was reliance on the HCAHPS survey as the primary metric for evaluating communication initiatives. The HCAHPS survey was created through a thorough development process and is widely highlighted due to its connection with hospital reimbursement under the Value-Based Purchasing program.^21^ Nonetheless, prior work documents that HCAHPS scores may be affected by factors other than physician communication and do not correlate with observed communication skills, such as demonstrating empathy; as a result, hospital medicine clinicians may not respond to feedback focused on HCAHPS results.^22–26^ Patient experience and communication are often used interchangeably, with an assumption that improving communication will inherently improve experience; however, these are distinct constructs, which may contribute to frustration when HCAHPS is used as the primary measure of communication quality.^26^

Several approaches could potentially address some of these concerns. Prior work showed that providing clinicians with real-time, daily feedback on HCAHPS performance (in addition to coaching) can improve HCAHPS performance, although this approach is resource-intensive and does not address more foundational concerns about the validity of HCAHPS questions themselves.^27^ Prior work by Roter and colleagues has highlighted the importance of systematic approaches to assessing clinician-patient communication quality using methods that extend beyond patient satisfaction measures.^28^ There are alternate, validated tools that may provide meaningful feedback to hospital clinicians; for example, the Communication Assessment Tool is a 14-item, psychometrically validated instrument that has been studied in hospital medicine settings.^29^ However, such tools may be resource intensive in that they require individuals to observe and provide feedback. Emerging technologies, such as artificial intelligence and ambient listening technologies, could potentially, with careful governance, provide clinicians with real-time, actionable feedback on written and verbal communication (e.g., use of jargon, checking for understanding) at scale. However, available research on these emerging technologies is primarily in the outpatient setting and assesses impacts to workloads as opposed to communication improvements.^30,31^

Another notable gap identified by participants was the lack of patient or caregiver input into initiative development and evaluation. Co-design methodologies that incorporate patient and caregiver perspectives into initiative development could help ensure that communication initiatives are meaningful.^32^ For example, Caven et al worked with both patients and clinicians to collaboratively design an initiative to improve serious illness conversations on general medicine wards, identifying optimal practices and perceived barriers from both patient and clinician perspectives.^33^ Our findings suggest that hospital medicine groups may need resources to effectively engage patients and caregivers beyond having a PFAC, given the perceived barriers to working with PFACs in initiative development.

Several of the identified barriers (including competing priorities, initiative fragmentation, variable leadership, and buy-in) are not specific to communication initiatives, but rather reflective of broader challenges in developing and implementing effective and sustainable changes in healthcare settings.^34^ There is evidence to suggest that multiple communication interventions, such as those intended to promote clinicians sitting at the patient bedside or those focused on patient counseling at discharge, are associated with improved patient outcomes^4,35,36^; nonetheless, participants described these initiatives being inconsistently implemented. It is also critical to understand the context in which initiatives are delivered, including how they integrate with existing workflows and may need to be adapted to meet the needs of hospital medicine clinicians. Furthermore, participants described a tension between efforts to track the use of communication best practices versus their quality or feasibility, and implementation science or quality improvement frameworks may be able to provide balanced feedback on both of these domains.^37^

This study has several limitations. Clinician participants were from academic settings, which may limit transferability. Participants (who were primarily physicians) may not be aware of concurrent, but important, communication initiatives being led by nursing or other interprofessional team members. Our recruitment through HOMERuN may have introduced selection bias, attracting participants with a strong interest in communication quality; however, multiple barriers to communication initiatives were identified. We included HOMERuN PFAC members in focus groups to obtain non-clinician perspectives and encourage discussion; however, these individuals represent a highly engaged population whose perspectives may not be representative of patient perspectives more broadly. We included probes in our interview guide to elicit patient and caregiver voices, but it is possible that PFAC members did not feel comfortable sharing their perspectives in this format. PFAC members did not participate in all focus groups, and this may have contributed to differences in discussion dynamics. Given our focus on institutional communication initiatives, we did not collect data on survey respondent race or gender. However, future initiatives should consider how these characteristics may influence communication experiences.^38,39^ Furthermore, we did not collect demographic characteristics for all focus group participants and therefore could not assess differences between survey respondents and non-respondents.

In summary, hospital medicine groups are engaged in numerous initiatives to improve communication with patients, but these efforts are undermined by fragmentation, inadequate feedback mechanisms, and competing priorities. Our findings highlight a need for coordinated strategies that align projects with clinical workflows, provide clinicians with actionable feedback, and incorporate genuine partnership with patients and caregivers. These approaches may enable health systems to develop sustainable communication initiatives that meaningfully improve patient experience, trust, and comprehension.

## Supplementary Material

Supplementary Materials

Additional supporting information can be found online in the Supporting Information section at the end of this article.

## Figures and Tables

**TABLE 1:** Identified themes, sub-themes, and representative quotes.^a^

*THEME 1: Hospital medicine communication initiatives vary widely in approaches and objectives*	
A. Numerous and diverse initiatives	“There is inpatient MyChart version that is specific to, you know, what’s going on during that stay. And so I know there’s a group working on, you know, what it is that patients want to see, how to make it easily, easily accessible at the bedside.” —Group 2 “It is a pretty rigorous communication training program. It was about an eight-hour session led by palliative medicine physicians with standardized patient actors and a lot of kind of evidence-based communication practices.” —Group 8
B. Varied objectives	“The idea is kind of empowering the patient and advising them what’s going on.” —Group 5 “There is definitely a large institutional push to continue to work on our HCAHPS provider communication scores.” —Group 8
C. Variable engagement across institutions	“We have had over the last decade a lot of initiatives …I don’t think we’re like actively following them.” —Group 1 “Been a lot of focus on this.… [describes multiple initiatives] …. And so, it’s been pretty robust for some time on those interventions.” —Group 5
*THEME 2: Foundational communication skills taught through formal and informal mechanisms, but may be siloed from other initiatives*	
A. Formal and informal initiatives with variable feedback	“The students get specific communication courses. The residents, it’s just, I think it’s pretty much built into their expectations.” —Group 2 “With the teaching hospitalists, we made an informal arrangement that at least a few patients every day, we would do full-fledged side rounding so have the trainees present the patients right next to the patient… and then we debrief after the visit and give them feedback on what went well, what could have done better.” —Group 4
B. Siloing between trainee and hospitalist initiatives	“It’s the sort of much, like overly blamed idea of siloing within academic medicine, right? But like, I don’t really know what they’re doing.” —Group 3 “My interactions with the trainees are actually pretty limited, so everything is a little bit like through the grapevine.” —Group 6
*THEME 3: Hospitalists perceive multiple barriers to improving individual communication behaviors*	
A. Lack of meaningful, accessible metrics & feedback	“People were not really able to be candid and honest with their colleagues with each other for fear of hurting their feelings.” —Group 5 “I guess I go back to the measurement and what feedback tools do we have to do to actually make people better, not to just measure these meaningless, probably mostly meaningless outcomes.” —Group 6
B. Communication behaviors challenging to change	“Getting experienced physicians to change their workflows and practice patterns is really, really hard. It’s much easier to teach students and residents because they’re open to that and experienced physicians aren’t.” —Group 3 “I think it’s a really hard skill to develop. I think most of what I’ve learned about it has come from my experiences at the bedside as a family member or as a patient myself.” —Group 6
C. Competing demands & priorities	“We’re so stuck in our team rooms…You can’t get away from your team’s chat, right? And it really hinders people going to the bedside.” —Group 1 “I will acknowledge that sometimes with the time pressure of like we need to see every patient and be able to present a plan as well to the case manager by a certain time means that I probably am forgetting the opportunity to give feedback at times.” —Group 4
*THEME 4: Systems-level factors make it challenging to effectively develop, evaluate, and sustain communication initiatives*	
A. Limited opportunities for patient involvement in initiative development	“We do have PFACs, but we’re only using them for kind of subspecialty style populations.” —Group 5 “I wouldn’t say it’s super patient-centric in my opinion; it is asking patients additional questions, but it’s not a patient-centric approach to thinking about this problem identification or needs assessment or how do we make this better.” —Group 6
B. Root causes of poor communication	“I think like some of the satisfaction things that have been designed, like sit down when you talk to the patient, like they’re relevant, but they’re like it’s not quite targeted to some of the challenges that we’re facing. …So, you know, do the things that we have to offer really address the root causes where people feel like they’re not, you know, trusting of their care team?”—Group 3 “So it’s not so much, you know, can I spend 10 or 15 minutes with the patient and family? I can. And, you know, educating them, but it’s not just me that has to do it. Now it’s, you know, the liver team or the infectious disease team or the cancer team, right?”—Group 2
C. Dispersed, fragmented initiatives with variable buy-in & leadership	“I think this subject is one that has suffered a little bit from the, like, division/institution who’s running the show problem that I think many of us face when we’re thinking about, like, divisional strategies.” —Group 3 “Most of these initiatives are rolling out from administrative departments that have no idea about the real world of rounding.” — Group 6 “Our hospital wants to increase its HCAHP scores. I honestly don’t care. My goal is to try just to improve patient care and patient understanding and build trust in our institution.” —Group 8

a Representative quotes in this table are from clinician focus group participants. PFAC member contributions are summarized separately in the “PFAC Member Contributions” section.

**TABLE 2:** Responses to survey questions (*N* = 26).

		*N* (%)
What is your role?	Attending Physician	24 (92)
	PFAC Member	2 (8)
What region is your practice located in?	Midwest	6 (23)
	Northeast	5 (19)
	South	4 (15)
	Southwest	3 (12)
	West	3 (12)
	Mid-Atlantic	3 (12)
	Pacific Northwest/Rocky Mountain	2 (8)
My institution has initiatives in place to improve patient-provider communication.	Yes	19 (73)
	No	3 (12)
	Unsure	3 (12)
	N/A	1 (4)
Does your institution have a Patient and Family Advisory Council?	Yes	18 (69)
	No	2 (8)
	Unsure	5 (19)
	N/A	1 (4)
What is the focus of ongoing initiatives in place at your institution to improve patient-provider communication?	Patient satisfaction	11 (42)
	Goal concordant care (e.g., assessing “What Matters Most)	3 (12)
	Other^a^	2 (8)
	Shared decision-making	1 (4)
	Discharge counseling	1 (4)
	Conflict de-escalation	1 (4)
	Disclosing medical errors/adverse events	1 (4)
	N/A; we don’t have any initiatives	5 (19)

a Both respondents indicated multiple initiatives at their institution.

## Data Availability

The data that support the findings of this study are available on request from the corresponding author. The data are not publicly available due to privacy or ethical restrictions.

## ELEVATOR PITCH

- We explored how academic hospital medicine groups are working to improve clinician-patient communication through focus groups with clinicians and members of a Patient and Family Advisory Council.
- Communication initiatives were common but highly variable, with clinicians reporting fragmented efforts, a heavy reliance on HCAHPS-based feedback, and limited patient involvement in program design.
- Improving communication may require coordinated strategies that fit into clinical workflows, provide actionable feedback to clinicians, and meaningfully partner with patients and caregivers.
