# Supplementary material for: Advancing clinician–patient communication in academic hospitals: Insights from a multisite rapid qualitative analysis
Source: J Hosp Med. Author manuscript; Available in PMC 2026 Nov 2. (PMC13726436; doi:10.1002/jhm.70525)
Supplement: Supplementary file 1 [file NIHMS2219257-supplement-Supplementary_Materials.docx]

**Supplement 1**

**Patient Communication Survey**

Please complete the survey below.

Thank you!

1. What is your role?

- Attending physician
- Advanced practice provider
- PFAC member
- Other (please describe): __________________________

2. What region is your practice located in?

- Pacific Northwest/Rocky Mountain Region
- West
- Midwest
- Southwest
- South
- Mid-Atlantic
- Northeast
- Missing

3. My institution has initiatives in place to improve patient-provider communication

- Yes
- No
- Unsure
- N/A

4. What is the focus of ongoing initiatives in place at your institution to improve patient-provider communication?

- Discharge counseling
- Medication counseling
- Patient satisfaction (e.g., HCAHPS)
- Goal concordant care (e.g., assessing “What Matters Most”)
- Advanced direction completion
- Shared decision making
- Conflict de-escalation
- Disclosing medical error / adverse events (e.g., Communication and Resolution programs)
- Ambient charting tools for inpatient care (e.g., DAX, Abridge)
- Other (please describe): __________________________
- N/A; we don’t have any initiatives in place

5. Does your institution have a Patient and Family Advisory Council?

- Yes
- No
- Unsure
- N/A

**Supplement 2:** Focus Group Guide

**HOMERuN Collaborative Session**: **Improving Patient-Provider Communication in the Hospital**

**Intro to Session Goals**

- **Review goal of session**: Goal is to characterize current efforts across hospital medicine programs to improve patient-provider communication, including initiatives targeted towards medical learners. We would also like to understand barriers to improving patient-provider communication in order to inform future efforts.

**Focus Group Questions:**

1. What work is your institution doing to improve patient-provider communication?
   - *Note:* In this context, “patient-provider communication” refers to initiatives focused on hospital medicine physicians and APPs
   - Probing questions: 1) What is the focus of this work (e.g., improving HCAHPS scores, other metrics or goals)? 2) In this work, what is the patient's role, or what is being done to include the patient/caregiver voices?
2. What work is being done related to learners/medical education and communication?
   - Probing questions: 1) Are any trainings required for all learners? 2) What role of trainees are targeted? 3) To what extent are patient/caregiver voices incorporated into these efforts?
3. What barriers or challenges has your division encountered in this space? Any barriers to implementing measures to improve patient-provider communication, etc?
   - Possible follow up if time: What initiatives are in place at your respective institutions to overcome these barriers and enhance patient-provider communication?  In what ways do these initiatives include patient/caregiver voices?

**Report back – Large group (~5-10 mins)**

- Question / discussion of findings
- Wrap up and thank you
